# Supplementary material for: IGF-1 Haploinsufficiency Causes Age-Related Chronic Cochlear Inflammation and Increases Noise-Induced Hearing Loss
Source: Cells. 2021 Jul 3;10(7):1686. doi: 10.3390/cells10071686 (PMC8304185; doi:10.3390/cells10071686)
Supplement: Supplementary file 1 [file cells-10-01686-s001.zip › cells-1276093-supplementary.pdf]

Supplementary Materials

Table S1. List of antibodies used in this study. IHF: Immunohistofluorescence, WB: Western Blotting.

Antibody	Manufacturer	Catalog Number	Species	Monoclonal or polyclonal	Use	Dilution
Synaptophysin	DAKO	A 0010	Rabbit	Pc	IHF	1:100
Neurofilament 200 KDa	Millipore	CBL212	Mouse	Mc	IHF	1:100
IBA1	Abcam	ab5076	Goat	Pc	IHF	1:100
Phospho p38 (Thr180/Tyr182)	Cell Signaling	9211	Rabbit	Pc	WB	1:1000
Phospho AKT (Ser473)	Cell Signaling	9271	Rabbit	Pc	WB	1:1000
Phospho-p44/42 MAPK (ERK1/2) (Thr202/Tyr204)	Cell Signaling	9101	Rabbit	Pc	WB	1:1000
Phospho-SAPK/JNK (Thr183/Tyr185)	Cell Signaling	4668	Rabbit	Pc	WB	1:1000
PI3K	In-house	-	Rabbit	Pc	WB	1:10000
p38α	Santa Cruz	sc-535	Rabbit	Pc	WB	1:4000
AKT1/2	Santa Cruz	sc-1619	Goat	Pc	WB	1:1000
p44/42 MAPK (ERK1/2)	Cell Signaling	9102	Rabbit	Pc	WB	1:1000
SAPK/JNK	Cell Signaling	9252	Rabbit	Pc	WB	1:1000

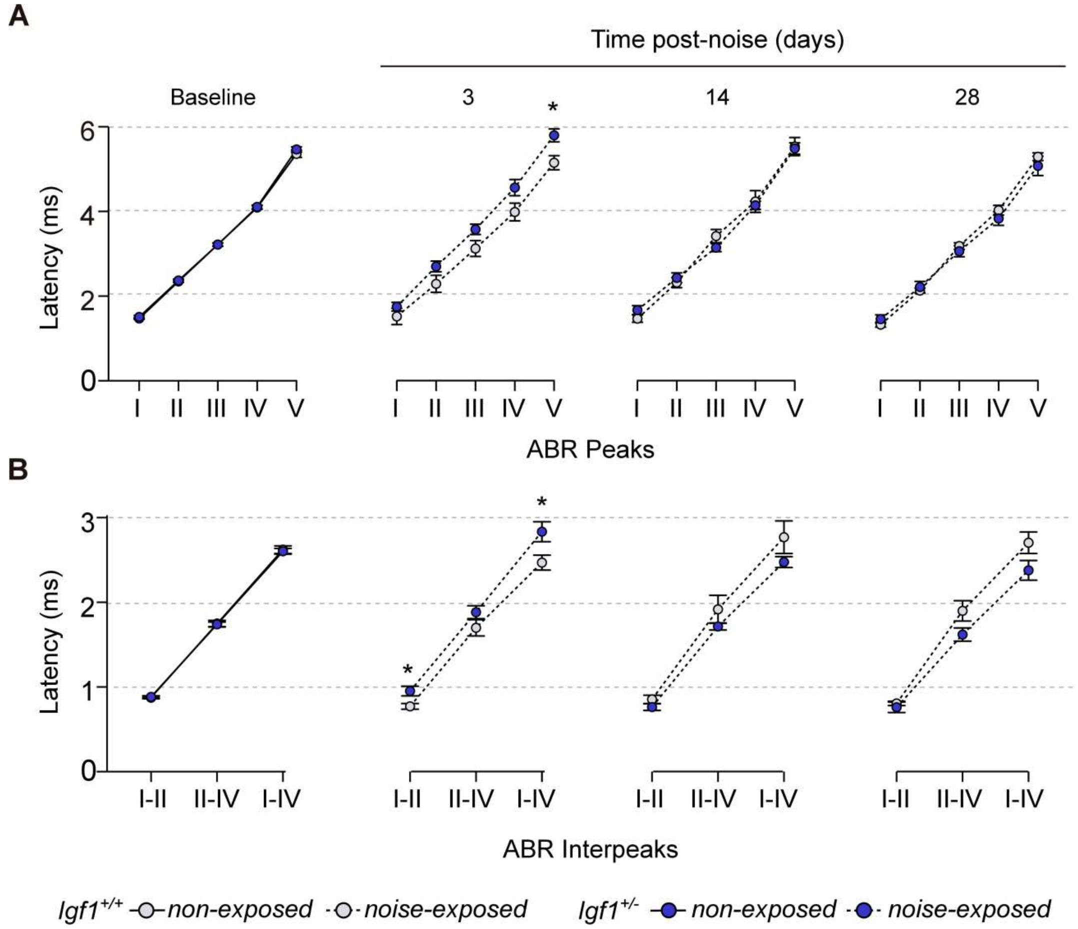

Figure S1. ABR latencies. ABR peaks I to V latencies (A) and interpeak latencies (B) (mean ± SEM) after click stimulation in *Igf1*$^{+/-}$ (n=9) and *WT* (n=7) mice before and 3, 14 and 28 days after noise exposure. Statistically significant differences were evaluated by Student's t-test (*p<0.05 between genotypes).
